# Surgical complications of cardiac implantable electronic devices: a systematic review and meta-analysis

**DOI:** 10.3389/fcvm.2025.1667583

**Published:** 2025-11-11

**Authors:** Ignacio Suárez-Paúl, Belén Redondo-Castro, José M. Gutiérrez Pastor, María José Membrive-Jiménez, Antonio Caballero-Mateos, José Luis Romero-Béjar, Guillermo A. Cañadas-De la Fuente

**Affiliations:** 1 San Agustín University Hospital, Department of Intensive Care, Andalusian Health Service, Linares, Spain; 2 Son Llàtzer Hospital, Balearic Islands Health Service, Palma, Spain; 3 Faculty of Health Sciences, University of Granada, Granada, Spain; 4 Faculty of Health Sciences of Ceuta, University of Granada, Ceuta, Spain; 5 Department of Gastroenterology, San Cecilio University Hospital, Andalusian Health Service, Granada, Spain; 6 Department of Internal Medicine, Gastroenterology Section, Santa Ana Hospital, Andalusian Health Service, Granada, Spain; 7 Department of Statistics and Operations Research, University of Granada, Granada, Spain; 8 Instituto de Investigación Biosanitaria (ibs. GRANADA), Granada, Spain; 9 Institute of Mathematics, University of Granada (IMAG), Granada, Spain; 10 Brain, Mind and Behaviour Research Center (CIMCYC), University of Granada, Campus Universitario de Cartuja, Granada, Spain

**Keywords:** morbidity, mortality, permanent pacemaker, postoperative complications, surgery

## Abstract

**Aim:**

To identify the most common complications that occur after the implantation of a permanent pacemaker, and to estimate their prevalence. Method: systematic review and meta-analysis. Selection criteria: quantitative primary sources, written in English, no age restriction, published between 1 January 2018 and 1 March 2025. Information sources: This 30 systematic review is based on a search of the PubMed scientific database, using descriptors from the MESH thesaurus in the following search equation: “Pacemaker, artificial AND Surgery AND Postoperative complications”.

**Risk of bias:**

Studies were assessed following the Mixed Methods Appraisal Tool (MMAT) guidelines. Results synthesis: descriptive analysis was used for the systematic review and random effects meta-analysis were performed.

**Results:**

Pacemaker implantation is a fairly common intervention, but it is not free of complications. The most frequent are pacemaker pocket infection, contusion or haematoma at the insertion site, painful shoulder and displacement of the generator or electrodes.

**Conclusion:**

It is important to take into account the patient's medical history and comorbidities, in order to match the device and its upkeep to the patient's characteristics, in order to minimise the risk of complications.

## 1 Introduction

A pacemaker (PM) is a medical device used to treat heart arrhythmia via the generation of regular electrical impulses, which replace the cardiac conduction system and ensure a synchronous, efficient heartbeat (1). A PM usually consists of two parts. A generator, which is implanted in the infraclavicular area of the non-dominant arm, and one or two electrodes, extend through a venous route from the generator site to the cardiac cavity. The decision to implant one or two pacing leads depends on the type of alteration that is to be treated. Depending on the number of cardiac chambers to be stimulated, the PM may be single or dual chamber. In addition, they may be used temporarily (in which case the generator remains outside the body and the electrode is inserted) or permanently (the generator is implanted beneath the skin) (2).

Permanent Pacemakers (PPM) are usually implanted under local anesthesia, with or without sedation, via the cephalic, axillary or subclavian veins. Dissection of the cephalic vein avoids the risk of pneumothorax that can occur with the subclavian puncture technique, but in order to perform it, the vein must be readily accessible and considerable surgical skill is required. Therefore, expert cardiac surgeons only employ this access route to implantation. Other surgeons with experience in the implantation of pacemakers, on the other hand, commonly apply the subclavian access technique. However, it is a blind implantation technique using anatomical references, which presents the risk of pneumothorax. The advantage of the axillary venous access technique is that it can be guided with ultrasound, one of the current gold standards for the management of venous access. After achieving access, a subcutaneous pocket is created within the infraclavicular area in which the pacemaker will be placed. Subsequently, its correct placement must be checked using an x-ray to ensure that both the leads and the generators are correctly located (3).

Cardiac pacing has become the common treatment for symptomatic bradycardia or high-grade atrioventricular block. An estimated 1.25 million permanent pacemakers are implanted worldwide each year (4). For example, 17,360 pacemakers were implanted in Spain in 2021. Most were first-time implantations, but generator and electrode replacements were also performed (5).

Permanent pacemaker implantation (PPI) is indicated in patients with bradyarrhythmia and/or heart blocks. These conditions are normally caused by dysfunction in the sinus node, where the impulse is generated, or by a failed conduction through the atrioventricular node (3).

Bradyarrhythmia and heart blocks are closely related and can occur simultaneously in some pathological conditions, depending on the characteristics of the patient and the clinical presentation (6, 7).

On average, among the patients with bradycardia attended in the Emergency Department, 15% present primary alterations of the conduction system, while the remaining 85% are distributed as acute coronary syndrome (40%), bradycardia secondary to medication (20%), metabolic causes (5%), implantable cardiac electronic device dysfunction (2%) and other causes (13%) (8, 9).

Regardless of the type of venous access employed, PPI is not without risk. Unfortunately, complications can occur during the process and in the postoperative period. Accordingly, postoperative care is very important to the detection and prevention of any such complications.

The primary care centre or the corresponding hospital service normally carries out the upkeep and monitoring of the PM (10).

Various complications may arise during or after this surgical procedure, but one of the most common is pocket haematoma, which usually occurs when haemostasis is incomplete or due to increased intrathoracic pressure. This circumstance can provoke bleeding at the electrode insertion point, especially among patients treated with anticoagulants. This haematoma usually resolves spontaneously, although it sometimes requires treatment. Another complication can be infection of the pacemaker; in this regard, the most frequent causative organisms are S. aureus, S. coagulase-negative, enterobacteriaceae, pseudomonas and fungi (11). When a pacemaker becomes infected, it is usually necessary to surgically remove the battery and electrode lead and to change their position. Lead displacement and/or perforation with system dysfunction is another relatively common complication. In addition, perforation of the pericardium can lead to pericardial effusion or cardiac tamponade. Other complications, which are less frequent but can have a severe impact, include damage of lead conductor, abnormalities in detection or capture 1, pneumothorax, haemothorax, air embolism, cardiac perforation, diaphragmatic stimulation and sepsis (3).

With respect to possible postsurgical infection, early infective endocarditis, caused by Staphylococcus aureus, is a major concern. This bacterium develops in the materials used to manufacture valve bioprostheses and Cardiac Implantable Electronic Devices (CIEDs). The growing presence of these devices has generated a corresponding increase in the prevalence of infective endocarditis, and hence an increased risk of embolism, stroke, sepsis and death.

Fungal infections are less common but can be very aggressive. The threat posed by coagulase-negative staphylococci (CoNS) infections is that they are found in the skin and can easily colonise external devices or infusion catheters. Other microorganisms, too, such as opportunistic pathogens, fungi and zoonotic bacteria, can adhere to the cardiac devices of the immunosuppressed patient, taking advantage of the patient's oropharynx as an entry route and causing insidious infections due to their slow growth (12).

In view of these considerations, the aim of this review is to identify the most common complications that may occur after the implantation of a PPM, and to estimate their prevalence.

## 2 Materials and methods

In this study, a systematic review and meta-analysis were conducted of the relevant scientific literature, in accordance with the provisions of the PRISMA statement (13).

### 2.1 Data sources and search equation

The data required for this analysis were obtained from the PubMed database, which was searched using Medical Subject Headings (MeSH) descriptors. The search equation used was “Pacemaker, artificial AND Surgery AND Postoperative complications”. The search for information ended on March 1, 2025.

### 2.2 Study selection

Selection criteria were applied to the results obtained after the initial search of the database. The PICO model was used to establish the eligibility criteria:

- P: Patients with heart rhythm disorders.
- I: Implementation of PM.
- C: (not applicable)
- O: Complications related to the implantation of a PM.

Inclusion criteria: full-text quantitative primary sources, written in English, no age restriction, published between January 1, 2018, and March 1, 2025.

Exclusion criteria: doctoral theses and articles unrelated to the focus of this study.

Once the aforementioned search criteria had been applied, the bibliography was selected in the following steps: reading the title and abstract, reading the full text and finally, and reverse search in the selected articles. The study selection process was peer-reviewed. For this purpose, two members of the team conducted the search and selection independently.

### 2.3 Study variables and data collection

To extract the data from each study, a data collection table was created with the following content: author(s), year of publication, country of study, sample characteristics and main results obtained regarding the surgical implantation of the pacemaker. These data were then subjected to a descriptive analysis (Table 1).

**Table 1:** Characteristics of the studies reviewed.

Author, country, year	Design	Sample	Risk factors	Epidemiology	Results	Le/DR
Alonso-Menchén et al. (23), Spain, (2024)	Retrospective	*N* = 6692	*Cutibacterium* spp*.*	25% of infective endocarditis was caused by a CIED	Molecular diagnostic tests have demonstrated useful and should be used routinely. A considerable percentage of cases develop complications, so cardiac surgery and removal of CIEDs play a key role in reducing mortality	3a/B
Boriani et al. (19), Italy, (2022)	Prospective	*N* = 2,675 patients - 1.658 patients in 11 sites of Northern Italy - 571 patients in Central Italy - 446 patients in Southern of Italy	Heart failure (27.7%), the use of oral anticoagulants (30.7%) and younger age	Final infection incidence: 1.1% patient-years	There were 28 (1.1%) CIED infections and 132 (5%) deaths, with 152 (5.7%) composite clinical event. Mean (SD) follow-up for CIED infection was 276.25 (143.61) days, while mean (SD) follow-up for composite clinical event was 273.57 (141.65) days	2a/B
				Rate of composite clinical events: 5.82% patient-years		
Carrión-Camacho et al. (43), Spain, (2019)	Prospective	*N* = 310 patients who received a PPM	Cardiovascular risk factors: hypertension, dyslipidemia and diabetes. Risk factors for device displacement: inadequate initial positioning, allowance of lead slack, and/or anchoring	PR without treatment = 3.22%. PR with treatment = 22.6%	Patients were followed up for 6 months to determine the most frequent major and minor complications. Patients with and without antithrombotics were compared to detect any differences between complications	2a/B
					The most frequent minor complication related to bleeding and the most recurrent complication was shoulder pain	
Carrión-Camacho et al. (29), Spain, (2020)	Prospective	*N* = 310 patients. 71 without antithrombotic therapy and 239 with antithrombotic therapy	Contusion (OR 2; 95% CI 1 to 3.8; *p* = 0.049), and minor complications, arm immobilization >24 h (*p*=<0.001) and contusion (*p* = 0.002)	PR with treatment = 25.10% PR without treatment = 14.08%.	Patients on antithrombotic treatment are more vulnerable. The most frequent major complications observed were electrode detachment and pneumothorax, to a similar degree in both groups. The most common minor complications were painful shoulder, haematomas and phlebitis. All of the deaths occurred in the antithrombotic therapy group. Within this therapy, the authors distinguished between oral anticoagulation, parenteral anticoagulation with heparin, and combined anticoagulation	2a/B
Dai et al. (17), China, (2019)	Prospective	*N* = 2,163 patients with CIEDs between 1988 and 2015	The analysis of risk factors did not reveal any statistically significant differences	–	Over a 28-year period, 62 cases of infection occurred in 59 patients, among which 3 patients had recurrent infections after removal and reimplantation of the device system	2a/B
El-Chami et al. (32), USA, (2019)	Prospective, non-randomised	*N* = 201 patients on haemodialysis at the time of PPM implantation	–	PR = 1.99%.	Patients with chronic kidney disease are more likely to require a PPM for the treatment of bradyarrhythmia, but its implantation increases the risk of complications. In addition to major complications (arrhythmias), other complications were observed: two cardiac effusions/perforation, a pseudoaneurysm, an infection of the abdominal wall, a metabolic acidosis problem, removal of the device and reduced blood pressure. All of these complications appeared within 25 days of the initial surgery. Among the 69 patients who were not undergoing dialysis, 76 complications were observed, including cardiac effusion or perforation, arterial injury, heart arrhythmia and adverse events at the puncture site	2b/B
El-Chami et al. (39), USA, (2019)	Clinical trial, randomised	*N* = 105 patients with a previous PPM infection	–	PR = 3.8% patients had major complications after PPM implantation without wires	There were no recurrent infections requiring removal of the Micra device. Leadless pacemakers help prevent infections and reduce endocarditis. The Micra leadless PPM is a safe and feasible option for patients with a history of CIED infection	1b/A
Glaser et al. (26), Sweden, (2021)	Prospective	*N* = 849 patients who underwent PPM implantation 30 days after TAVI	–	PR = 26.03%.	Patients who undergo PPM implantation after TAVI are at greater risk of heart failure, hospitalisation and mortality. No relationship with endocarditis was observed	2a/B
Guan et al. (22), China, (2018)	Retrospective	*N* = 1259 patients who received a PPM	Male sex, diabetes, ESKD, duration of operation, PPM replacement and low central volume	PR = 1.90%.	Patients with endocarditis related to pacemaker lead infection, when the leads have not been removed, present a higher mortality rate. This highlights the importance of preventing infection during the perioperative period and surgical procedures. This is one of the main causes of severe systemic infection and even septic shock	3a/B
Hasan et al. (42), Germany, (2022)	Retrospective	*N* = 123693 CVC = 75.25 (60.8%) S*P* = 48.442 (39.2%)	–		There were significantly less permission/postoperative complications in the CVC group compared to the SP Group (2.49% compared to 3.64%, *p* = 0.0001, or 1.47; 95% IC 1.38–1.57)	3a/B
Imberti et al. (18), Italy, (2023)	Prospective	*N* = 838. PPM = 569 ICD/CRT = 269	End-stage chronic kidney disease requiring dialysis and corticosteroid therapy		Only 5 patients had implementation related (0.6%). Positive blood cultures in two patients (Staphylococcus aureus and Escherichia coli). None died for this reason	2a/B
Jacheć et al. (20), Poland, (2025)	Retrospective	*N* = 3487 *n* = 2,640 transvenous lead extraction (68.62%). *n* = 361 isolated pocket infection (9.38%). *n* = 472 pocket infection complicated by infective endocarditis (12.27%). - *n* = 374 lead-related infective endocarditis (9.73%)	Common risk factors: male gender, presence of an ICD lead, presence of an abandoned lead, last CIED-related procedure, and reintervention rate. Isolated pocket infection: lead abrasion. Pocket infection complicated by infective endocarditis: diabetes, renal failure, immunosuppressive therapy, and multiple bypasses Lead-related infective endocarditis: diabetes and intra-cardiac lead abrasion		Mortality in infectious patients was almost 10 times higher than mortality in non-infectious patients (7.49% vs. 0.83%; *p* < 0.001). Comparing the survival of patients with pocket infections versus patients with electrode-related infections, it was better in the former group (46.82% vs. 37.70%; *p* < 0.001) although it was also worse at the one-year follow- up [median 1,828 (815–3,139) days]. When comparing rates at longer follow-ups, both groups were equal	3a/B
Jiménez et al. (41), Spain, (2019)	Prospective	*N* = 240 patients were randomized to receive CIED implantation by the fluoroscopy-guided axillary vein access vs cephalic vein access	–		The success rate of the randomized venous access was superior in the axillary group than in cephalic (98.3% vs 76.7%, *P* < 0.001). Time to access (6.8 ± 3.1 min vs 13.1 ± 5.8 min, *P* < 0.001) and implantation duration was significantly shorter in the axillary group than in the cephalic group (42.3 ± 11.6 min vs 50.5 ± 13.3 min, *P* < 0.001). There was no difference in the incidence of complication and inter-operator success rate, complications rate and time to access	2a/B
Jing et al. (30), China, (2020)	Retrospective	*N* = 124 patients who received a PPM	Older age, high BMI, smoking history, poor nutritional status, and decreased platelet counts	PR = 8.06%.	The incidence of complications was 8.06% (10 cases), among which were haematoma (the risk was greater in anticoagulated patients), infection and venous thrombosis. Complications were more frequent among patients with comorbidities. The risk of pocket haematoma is increased by factors such as incomplete intraoperative haemostasis and bleeding from small arteries. The risk of DVT is increased by hypercoagulability and reduced physical activity (especially in older patients)	3a/B
Jiwani et al. (37), United States of America, (2025)	Retrospective	*N* = 10,342 ESKD patients with a CIED	Higher BMI (aHR, 1.01; 95% CI, 1.01–1.02), younger age (aHR, 0.96; 95% CI, 0.96–0.97) and shorter duration of dialysis (aHR, 0.94; 95% CI, 0.89–0.98)	PR = 6.1% of CIED infections	CIED infections are common in patients with end-stage renal disease (ESKD) and are associated with high 1- and 3-year mortality	3a/B
Kaplan et al. (27), United States of America, (2019)	Retrospective	*N* = 67 patients who underwent PPM implantation after TAVI	Hypertension and postballoon dilation	PR = 13.43%.	TAVI may present complications that require the insertion of a PPM. When this occurs, some patients may become dependent on the PPM. This dependency is more common in patients with self-expanding valves and balloon dilation	3a/B
Lin et al. (24), USA, (2021)	Retrospective	*N* = 233 patients who underwent CIED removal due to complications	–	PR bacteraemia = 54.51%. PR pocket infection = 45.49%	In the group with bacteraemia, S. aureus was the most prevalent organism, followed by E. fecalis, S. coagulase negative and other streptococcus species. Six patients with bacteraemia, all in the delayed extraction group, had infection by multiple organisms. In the pocket infection group, the most common organism was S. coagulase negative, followed by S. aureus, Gram negative bacteria, enterobacteria and fungi	3a/B
				Mortality rate of isolated pocket infection: 0.9%. Mortality rate in the late extraction group: 1%	Delayed removal of the CIED due to infection is associated with major complications. Among the delayed extraction groups, mortality was higher among patients with bacteraemia than in those with pocket infection	
Ma et al. (36), China, (2020)	Retrospective	*N* = 130 cases, 60 of whom presented DVT and 70 without complications	–	PR = 46.15%.	DVT can appear after PPM implantation. Relevant factors include a strong inflammatory system response and an increase in coagulation factors (hence, decreased fibrinolysis). The implanted electrodes can damage the blood vessels, disrupting the inflammatory system and the release of coagulation factors, which can aggravate or generate thrombi	3a/B
Markos et al. (35), Ethiopia, (2024)	Retrospective	*N* = 118 patients operated between 2017 and 2022	Age over 70, being female, PPM implanting team, and follow-up compliance		Most frequent comorbidity is hypertension. With an average follow -up of 3.92 ± 1.94 years, 15.3% of patients presented complications. The pneumothorax, the infection of the bag and the detachment of the electrode were the most frequent complications, which occurred in 2.54% of the patients each	3a/B
Nasir et al. (33), Ethiopia, (2024)	Retrospective	*N* = 182 who underwent PPM implantation	Advanced age, type of pacemaker (dual-chamber), lead displacement, and heart failure	PR = 26.4%	The three most frequent complications were the detachment of the electrode, which affected 6.6% of the patients, PPM induced tachycardia, which affected 5.5% of the patients, and the early exhaustion of the battery, which affected the 5.5% of patients. Patients with a higher Charlson comorbidity index before PPM implant (AOR 1.2, 95% IC 1.1–1.8, *p* = 0.04), presence of complications (AOR 3.5, 95% IC 1.2–10.6, *p* < 0.03) and Class III or IV of the New York Heart Association (NYHA) (AOR 3.3, 95% IC 1.05–10.1, *p* = 0.04) were associated with mortality. The most common comorbidity was hypertension (62.1%), followed by diabetes mellitus (47.8%)	3a/B
Oh et al. (21), USA, (2019)	Prospective	*N* = 433 patients	–	PR = 4.85%	S. Aureus and coagulase negative cause 60%–80% of PPM infections, while enterococcus is uncommon. Most infections are of haematogenous origin and late, appearing after 12 months	2a/B
Olsen et al. (16), Denmark, (2022)	Prospective	*N* = 84429 patients undergoing CIED implantation or reoperation	Pocket CIED infection: male sex, young age, CRT systems, CIED reoperations, systemic lupus erythematosus, previous valvular surgery, and recent use of dicloxacillin. Systemic CIED infection: male sex, young age, CRT systems, severe renal insufficiency/dialysis, prior valvular surgery, dermatitis and usage of insulin		A total of 1,556 CIED explanations were classified as either pocket (*n* = 1,022) or systemic CIED infection (*n* = 534). Severe renal insufficiency/dialysis (HR: 2.40, 95% CI: 1.65–3.49), dermatitis (HR: 2.80, 95% CI: 1.92–4.05), and previous valve surgery (HR: 2.09, 95% CI: 1.59– 2.75) were associated with the highest risk of systemic CIED infections	2a/B
Ponta et al. (25), Italy, (2025)	Retrospective	*N* = 232 patients with CIED infection. Among them, 147 patients started empirical antibiotic treatment	–	PR = The treated patients (147) presented the following figures: 90.5% with pocket infection, 29.9% with electrode infection; 6.1% with valvular endocarditis. At six months, 7.3% had recurrent CIED infection; 7.3% died	Experimental antibiotic treatment with daptomycin in combination with ceftriaxone in patients with CIED infection decreased recurrence and mortality rates at 6 months. In addition, they did not have any significant drug-related adverse events. Specifically, high doses of these drugs represented a safe and effective option for the empirical treatment of CIED infections	3a/B
Popiolek-Kalisz et al. (38), Poland, (2025)	Retrospective	*N* = 1,673 patients who underwent CIED implant	Number of leads		Comparison of the incidence of complications between subgroups according to the number of shunts affirms that there is a higher risk in patients with a greater number of shunts (*p* < 0.001). Subgroup analysis relative to the type of complication revealed the predictive value of the number of shunts for pneumothorax (*β* = 0.89; *p* = 0.04) and shunt dislocation (*β* = 0.67; *p* = 0.01)	3a/B
Shokr et al. (31), USA, (2019)	Prospective	*N* = 1,304,376 hospitalised patients, of whom 56,258 had chronic thrombo-cytopaenia	–	PR = 4.31%.	The patients with chronic thrombocytopaenia had higher in-hospital mortality after cardiac procedures, and increased complications. The incidence of cardiac tamponade, haemorrhagic stroke and ischaemic stroke was similar in both groups. The risk of in-hospital mortality in patients with chronic thrombocytopenia was 1.5 times higher	2a/B
Táborský et al. (34), Czech Republic, (2023)	Retrospective	*N* = 114,000 pacemakers were implanted between 2010 and 2021			The relative survival observed at age 5 was 88.6% (global survival of 60.6%) and survival relative to 10 years was 75.9% (global survival of 32.7%). The causes of death varied according to the patient's age. In the 2010–2019 period, the most frequent cause of death in people with MP implanted were cardiovascular diseases In the 2020–2021 years affected by the Covid-19 Pandemia, cardiovascular diseases remained the most frequent cause of death in people with MP implanted The proportion of deaths from diseases of the circulatory system increases with age, while the proportion of cancer deaths decreases	3a/B
Toon et al. (40), 2025, United Kingdom, (2025)	Retrospective	*N* = 81 patients	–	PR = 2% in the group that was discharged the same day. PR = 7% in the group that stayed overnight for observation	CIED implantation without electrodes was successful and decreased the rate of postsurgical complications. Major complications: 1 patient had pulmonary embolism for 2 weeks; 1 patient had detachment of the Micra: and 1 patient developed arterio- venous fistula requiring surgery. Minor complications: 3 patients with bleeding/hematoma at the puncture site; 1 patient with infection at the puncture site	3a/B
Wolfes et al. (28), Germany, (2024)	Retrospective	*N* = 1038 TAVI patients	–	PR = 11.5%	Despite the risk of complete persistent heart block in patients undergoing TAVI, 19 patients underwent cardiac pacing for borderline cardiac anomalies (A-V block)	3a/B

AOR, adjusted odds ratio; A-V, atrial-ventricular; aHR, adjusted hazard ratio; BMI, body mass index; CI, confidence intervals; CIED, cardiac implantable electronic devices; CRT, cardiac resynchronization therapy; CVC, cephalic vein cutdown; DR, degree of recommendation; DVT, deep vein thrombosis; ESKD, end-stage kidney disease; ICD, implantable cardioverter defibrillator; LE, level of evidence; PPM, permanent pacemaker; PR, prevalence rate; SD, standard deviation; SP, subclavian puncture; TAVI, Transcatheter aortic valve implant.

### 2.4 Critical reading and scientific evidence

The quality of each study was evaluated in accordance with the levels of evidence and grades of recommendation stipulated by the Oxford Center for Evidence-Based Medicine (OCEBM) (14).

### 2.5 Risk of bias

Studies were assessed following the Mixed Methods Appraisal Tool (MMAT) guidelines, so that any potential biases and limitations could be discussed (15).

### 2.6 Meta-analysis

The meta-analysis was performed with the software Stats Direct. Two random effect proportion meta-analysis were performed. I2 was used for heterogeneity and Egger bias was used for publication bias.

## 3 Results

### 3.1 Search results and study characteristics

**Figure 1:**
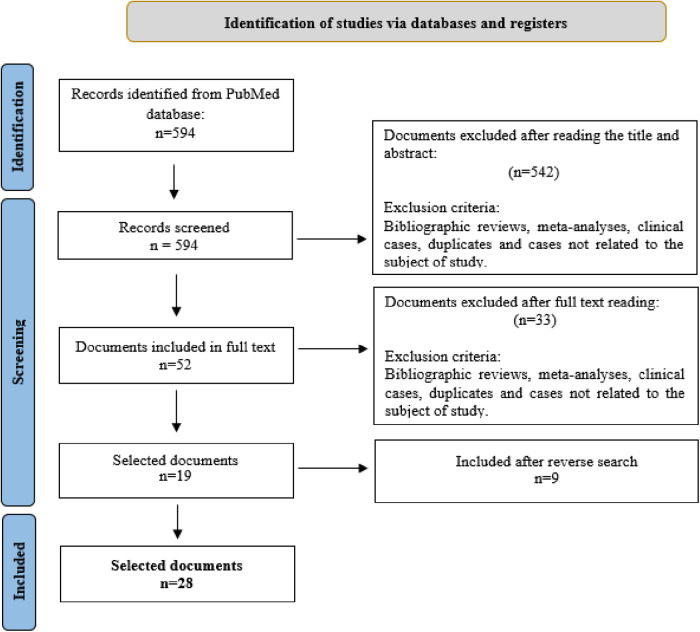
Selection of documents included in the review. From: Page et al. (13).

The initial literature search obtained 594 articles. After removing duplicates and those articles deemed irrelevant following a reading of the title and abstract, 52 papers remained for a full-text reading. Of these, 33 were discarded because they did not address the specific topic of this review. However, a reverse search produced 9 additional papers. Hence, the final sample selected for review was composed of 28 articles. The article selection process is summarised in Figure 1.

Most of the studies considered were observational in design, although one paper described a clinical trial. Various countries of origin were represented; the majority of studies were published in Spain, China and the USA.

### 3.2 Infection following pacemaker implantation

Research by Olsen et al. (16) conducted in 84,429 patients undergoing 108,494 CIED surgeries highlights the varying risks of infection associated with different cardiac devices. This study revealed that pocket infections accounted for about 66% of CIED infections, while systemic infections accounted for approximately 33%. Furthermore, PPM implantations were found to have a longer median time to CIED systemic infection (640 days) compared to Cardiac Resynchronization Therapy with a Defibrillator (CRT-D) patients (197 days).

The study by Dai et al. (17) involving 2,163 CIED patients from 1988 to 2015 reported an infection rate of 4.2 per 1,000 person-years, with variations by device type. PPM had an infection rate of 2.9 per 1,000 person-years, implantable cardioverter defibrillators (ICDs) had an infection rate of 8.4 per 1,000 person-years, and cardiac resynchronization therapy (CRT) devices had an infection rate of 11.0 per 1,000 person-years. Furthermore, infection rates were significantly higher in both the ICD and CRT groups compared with PPM patients (CRT vs. PPM; *p* = 0.002; ICD vs. PPM; *p* < 0.001). In the study, 66.1% of infections were pocket infections, while 33.9% were bloodstream infections, including cases of endocarditis.

Imberti et al. (18) monitoring 838 patients intervened in September 2016 and August 2020. A group of 569 patients implanted a PPM and another group of 269 patients was implanted with an implantable cardioverter (ICD)/cardiac resynchronization therapy (CRT). The ICD/CRT group was checked at 6 months (in them antibiotic wrapping) and the PPM group at 12 months. Only in 5 patients was PPM implantation associated with infection (0.6%), after a median follow-up of 42.3 months (30.2–56.4). There were positive blood cultures in two patients (Staphylococcus aureus and Escherichia coli). None died for this reason.

The study by Boriani et al. (19), which enrolled 2675 patients across 18 active sites, further examined the risk factors and outcomes associated with CIED infections. PPM were the most common devices implanted (66.7%), followed by implantable cardioverter defibrillators (ICD) and cardiac resynchronization therapy devices (CRT-P and CRT-D). Key risk factors for infection were diabetes, severe kidney disease, and hospital-acquired infections (HAI), which were more prevalent among patients who had undergone device revision, upgrading, or reimplantation procedures. The study also developed a clinical risk score, the RI-AIAC Infection Score, which identified patients at higher risk of infection based on these factors.

Jacheć et al. (20) state that diabetes and intracardiac abrasion predispose patients to electrode-related infectious endocarditis. They also say that the presence of multiple electrodes and Staphylococcus aureus in the peritoneal cavity culture are risk factors for the spread of infection. Between the two, electrode-related infectious endocarditis had a worse outcome.

Oh et al. (21) conducted a prospective study with 433 patients, of whom 21 had enterococcal infection, 11 had fever and 7 had sepsis. Only 14 had had their pacemakers removed. The authors reported that enterococcus represented only 4.8% of all infections diagnosed during the three-year study period, a much lower proportion than was recorded for Staphylococcus aureus and CoNS, which jointly account for 60%–80% of all cardiac device infections. Finally, for the majority of patients in this study, the infection appeared more than 12 months after implantation of the CIED (i.e., delayed extraction), which suggests that it probably arose in the context of a transient bacteraemia.

### 3.3 Infections from failure to remove electrodes or CIED

Infective endocarditis is a rare but potentially serious condition. Guan et al. 22 conducted a three-year follow up of 1,259 patients in whom a PPM had been implanted. These authors emphasised the importance of preventing infection during surgical procedures and throughout the perioperative period. In addition, the mortality rate of patients with infection in whom the leads were not removed from the cardiac device was reported to be 66%.

In a similar study with 6,692 patients, Alonso-Menchén et al. (23) identified CIEDs as the cause of 25% of infective endocarditis. The median time from CIED implantation to IE diagnosis was 19 (IQR: 2–90) months and 46 (IQR: 24–95) months, respectively. This diagnosis was made possible by the new Duke-International Society for Cardiovascular Infectious Diseases criteria.

In another study, Lin et al. 24 analysed all extractions of infected CIEDs performed at their medical centre from 2006 to 2019. The patients were divided into two groups according to the presence or otherwise of bacteraemia or pocket infection. In-hospital morbidity and one-year mortality were evaluated for early vs. delayed treatment. Of 233 patients who underwent CIED removal, 127 had bacteraemia and 106 had pocket infection. In both groups, delayed CIED removal was associated with higher one-year mortality and worse outcomes. Of the patients with bacteraemia in the delayed extraction group, 11 died during hospitalisation compared to none in the early extraction group. Of the patients with isolated pocket infection, one in the delayed extraction group died from cardiac arrest, compared to none in the early extraction group. Accordingly, the authors emphasised the importance of the early detection of infection and removal of the CIED lead.

According to Ponta et al. (25), they carried out a study with experimental treatment in which they prescribed antibiotics for patients admitted with CIED-associated infection. This experimental treatment was carried out with daptomycin combined with ceftriaxone, reducing recurrence and mortality rates at 6 months. In addition, there were no significant adverse effects related to pharmacological treatment.

### 3.4 Cardiovascular complications associated with cardiac implantable electronic devices

According to Glaser et al (26) 9%–26% of patients who undergo transcatheter aortic valve implant (TAVI) require PPI, placing them at greater risk of heart failure, hospitalisation and mortality. In their study, of the patients in the pacemaker group who died, 51.4% presented cardiovascular causes. However, no significant association was found between pacemaker implantation and these deaths.

Kaplan et al. (27) studied 67 patients who received PPI after TAVI. A potential complication arising from TAVI is the development of complete heart block or other conduction disorders, requiring the implantation of a pacemaker. On the other hand, fewer than half of patients who receive a pacemaker after TAVI are pacemaker-dependent at 30 days after the intervention, and this level of dependence decreases in the first year. Of these 67 patients, 27 were pacemaker-dependent within 10 days and nine were dependent in the first year. This dependency is more common in patients given self-expanding valves and balloon dilation.

Wolfes et al. (28) retrospectively analysed patients who underwent TAVI and PPI. Although pacemaker implantation was safe; there were patients in whom atrioventricular block persisted. For this reason, they required high amounts of pacing after 24 h. This problem persisted in some patients even six weeks after the procedure.

### 3.5 Permanent pacemaker implantation and comorbidities

Carrión-Camacho et al. (29) compared two groups of patients implanted with a PPM, one treated with antithrombotic therapy (77%) and the other did not receive this therapy (23%). There was observed to be greater frailty in the group with antithrombotic therapy, due to the associated comorbidity. The most common major complications in the antithrombotic therapy group were pneumothorax and electrode dislodgement. Among the minor complications encountered were uncomplicated haematomas (24.7% vs. 15.5%), painful shoulder (more common in the group without antithrombotics, with a prevalence of 28.2% vs. 15.9%, respectively), and phlebitis, which had a similar prevalence in both groups.

Jing et al. (30) noted that anticoagulated patients implanted with a PPM have an increased risk of pocket hematoma. Excessive use of heparin or the combined use of heparin with aspirin may increase this risk. Therefore, interrupting the use of anticoagulant drugs before surgery might be advisable.

Shokr et al. (31) reported that patients with chronic thrombocytopaenia was associated with a higher risk of complications after TAVI, PPM and other cardiac interventions. Haematomas and post-operative bleeding were the most frequent complications, although cardiac tamponade and stroke also occurred. Also, presented greater in-hospital mortality after cardiac surgery, El-Chami et al. (32) in a study of haemodialysis patients found that permanent atrial fibrillation with bradyarrhythmia was the main indication for pacemaker implantation. Following this, the main complications were related to device problems, intermittent capture loss, dislodgement without embolisation, and device embolisation during attempted implantation. Therefore, considerations such as comorbidities and dialysis access must be considered. In patients undergoing haemodialysis, wireless stimulation helps prevent device infection and is a good option.

Nasir et al. (33) conducted a retrospective study in Ethiopia where they reviewed patients who underwent PM implantation from October 2023 to January 2024. 26.4% suffered postoperative complications. The most common complications were lead dislodgement, PPM-induced tachycardia and early battery depletion. Patients with a higher Charlson comorbidity index before PPM implantation, presence of complications, and New York Heart Association (NYHA) class III or IV were associated with mortality. The most common comorbidity was hypertension (62.1%), followed by diabetes mellitus (47.8%). In the study by Táborský et al. (34), a significant number of patients had comorbid conditions: 29.1% had diabetes mellitus, 84.4% hypertension, 18.7% a history of ischemic heart disease, and 13.8% a history of heart failure. The study also assessed long-term survival after first pacemaker implantation. The overall 5-year survival rate was 60.6%, while the 10-year survival rate was 32.7%. Relative survival, which compares observed vs. expected survival in the general population, was 88.6% at 5 years and 75.9% at 10 years. Survival outcomes varied by pacemaker type, with dual-chamber pacemaker recipients showing the best survival rates. The leading cause of death among pacemaker patients was cardiovascular disease, accounting for 62% of deaths between 2010 and 2019.

The study by Markos et al. (35) included 118 patients, with a mean follow-up of 3.92 ± 1.94 years. The most frequent comorbidity was hypertension (64.2%). In addition, many patients (53.4%) had symptoms such as dizziness, syncope and mild dyspnea prior to pacemaker implantation. The observed complication rate was 15.3%, with factors such as age, sex, preimplantation symptoms and adherence to follow-up associated with a higher risk of complications.

Ma et al. (36) conducted a prospective study of 130 cases of pacemaker implantation, divided into two groups: the patients who had presented deep vein thrombosis (DVT), and those who had not. Important factors in the development of DVT include a strong inflammatory reaction and an increase in the coagulation factors (plasminogen activator inhibitor and thrombin-activated fibrinolysis inhibitor) that decrease fibrinolytic activity. Pacemaker implantation alters the release of coagulation factors and promotes additional thrombus formation, because the implanted electrodes damage the blood vessels. This factor must be taken into account because disruption of the inflammatory system can lead to or aggravate thrombus formation. If this is detected, the risk of further development might be reduced by considering the relationship between inflammation and DVT.

Jiwani et al. (37) CIED infections are common in patients with end-stage kidney disease (ESKD) and are associated with high 1- and 3-year mortality. Of the patients who developed infection, 50.71% underwent electrode removal. Removal is associated with some improvement in survival. However, it is currently performed in only half of the patients due to high infection rates and consequent mortality.

### 3.6 Complications related to electrodes and wires

Popiolek-Kalisz et al. (38) in their investigation of 1,673 patients stated that the number of electrodes was the main cause of early complications after CIED implants. More specifically, electrode dislocations and pneumothorax occurred.

El-Chami et al. (32) observed that patients with chronic kidney disease are at increased risk of dialysis access-associated bacteremia. In addition, venous stimulation by pacemaker leads may cause stenosis of the subclavian vein in up to 70% of patients. They therefore deduced that pacing with a wireless pacemaker seems to be the best option.

El-Chami et al. (39) compared the outcomes obtained for 105 patients with previous infection who underwent an attempted implantation of a leadless pacemaker (Micra), during the 30 days following removal of the previous implantation. This study recorded only six major complications, in four patients, related to this procedure. No recurrent infections requiring removal of the Micra device were recorded during the follow-up period. Significant characteristics of this type of device include its intracardiac location, the small surface area presented and the tendency to encapsulation, which could be an advantage for patients at risk of recurrent infection.

According to Toon et al. (40), hospital admission for CIED procedures can place a burden on medical resources. Their study suggests that outpatient surgery for MICRA leadless pacemaker implantation can be performed safely in a carefully selected population. The complication rate was minimal, ranging from 2% to 7%.

### 3.7 Comparison of complications between different access techniques for pacemaker implantation

In a study by Jiménez et al. (41) 240 patients were randomly assigned to undergo pacemaker implantation using fluoroscopically guided axillary venous access (axillary group, *n* = 120) or cephalic venous access (cephalic group, *n* = 120). The study showed that the axillary group had a significantly higher venous access success rate compared to the cephalic group (98.3% vs. 76.7%, *p* < 0.001). Access time and implantation duration were significantly shorter in the axillary group (6.8 ± 3.1 min and 42.3 ± 11.6 min, respectively) compared with the cephalic group (13.1 ± 5.8 min and 50.5 ± 13.3 min, *p* < . 001). Although complications were more frequent in the cephalic group (9.1%) compared with the axillary group (5%), the difference was not statistically significant (*p* = 20). These findings suggest that axillary venous access may be more efficient and have a higher success rate without increasing the risk of complications.

In a similar study, Hasan et al. (42) who analyzed complications related to central venous access (CVC) and subclavian access (SP) in 123,693 patients. In this study, CVC was associated with a lower complication rate (2.49%) compared with SP (3.64%) (*p* < 0.0001). The odds ratio for complications was 1.47 (95% CI: 1.38–1.57, *p* < 0.001) for SP compared with CVC. Lead dislocation was the most common complication in both groups, while pneumothorax was five times more frequent in the SP group compared with the CVC group (0.85% vs. 0.15%). The comparison between the techniques further highlights the potential benefits of choosing axillary or CVC access over more commonly used approaches such as subclavian or cephalic access in terms of efficiency and reduced risks of complications.

### 3.8 Other potential complications after permanent pacemaker implantation

Carrión-Camacho et al. (43) performed a prospective study in which a sample of 310 patients were fitted with a PPM, during a 12-month period. These patients were followed up for six months.

The most frequent major complications were pneumothorax (3.87%) and cable detachment (8.39%), while minor complications included superficial phlebitis (12.9%), uncomplicated haematomas (22.58%)—more frequent in patients who had received previous antithrombotic treatment—and shoulder pain, which was more recurrent in the group that had not received antithrombotics.

In their study of 124 patients aged 30–86 years, Jing et al (30) recorded a complication rate of 8.06%. The complications, which included haematomas of the pacemaker pocket, infection and venous thrombosis, were more frequent in patients with comorbidities, in older patients and when the blood platelet count was reduced. The risk of pocket haematoma is increased by incomplete intraoperative haemostasis, bleeding from small arteries into the generator sac cavity, inadequate generator sac size and the presence of multiple punctures. Elderly patients, furthermore, are more likely to have hypercoagulability and reduced physical activity, which can result in postoperative complications such as venous thrombosis.

### 3.9 Meta-analysis

**Figure 2:**
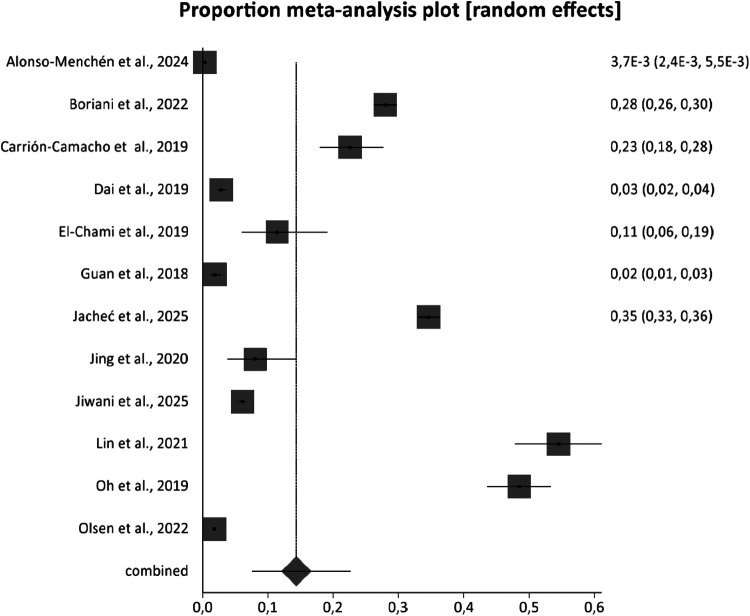
Rate of infection related to pacemaker implantation.

According to our meta-analysis of a sample of *n* = 112,252, the rate of infection related to pacemaker implantation was 14.4% (95% C.I. 7.65%–22.66%) with an *I*^2^ value of 99.8% (Figure 2).

**Figure 3:**
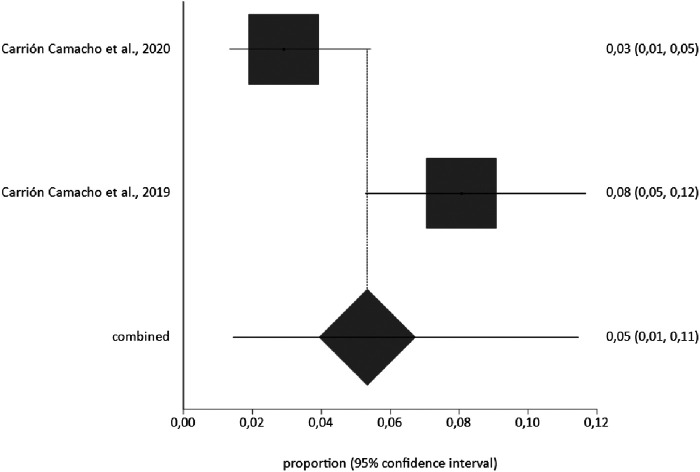
Prevalence of pneumothorax as a complication of pacemaker implantation.

The corresponding meta-analysis for the prevalence of pneumothorax as a complication of pacemaker implantation, with a sample of *n* = 620, produced a rate of 5% (95% CI 1%–11%), with an *I*^2^ value of 87.2% (Figure 3).

## 4 Discussion

A pacemaker is a medical device to manage heart arrhythmia by means of electrical impulses. It is inserted within the patient in a surgical intervention which, like any surgery, is subject to risks and potential complications.

Currently, survival rates have increased considerably, with new diseases appearing and the incidence of other known diseases increasing. In the case of cardiac arrhythmias, and specifically bradyarrhythmias, there has been a proportional increase in line with survival rates. Concomitant risk factors and the onset of chronic diseases, which lead to increased medication use, cause an increase in the incidence of bradyarrhythmias. As a result, the implantation of an ICD is becoming increasingly necessary, also increasing the complications associated with these devices (44).

One of the most common problems that may arise following PPI is that of infection, either localised within the pacemaker pocket or in the form of bacteraemia. However, rates of infection are low in developed countries, due to improvements in sanitation, sterilization and implantation techniques (45). On the other hand, rising demand for the intervention means that, worldwide, cardiac pacemaker infections are increasing due to inadequate knowledge about the risk factors that contribute to their occurrence (46).

The majority of CIED infections are caused by S. aureus and so the approach to prevention must be multidisciplinary. If the device is infected, it must be removed and the wound allowed to close by secondary intention until the infection is under control, and a new device can be implanted (45).

Most ICDs use wires that connect the device to the heart. The presence of a foreign body is a constant concern due to the risk of infection or malfunction of the electrodes, so removal is usually the first option (47, 48). In fact, proper removal of electrodes increases the success rate and safety, allowing for subsequent reimplantation (49). Conversely, delaying removal increases the mortality rate due to infected CIEDs (50).

High doses of anticoagulants or their combined use with antiplatelet agents can cause complications such as pocket haematoma. However, when anticoagulant therapy is necessary, despite a higher rate of haematomas, there is less chance of suffering a stroke or other complications (51, 52). It should also be noted that haematomas are more common in older patients, as they are more likely to have hypercoagulability and reduced physical activity, in line with frailty syndrome (44).

In addition to CIED, other types of cardiac intervention such as TAVI may be performed. Certain complications may arise following TAVI implantation. One of these is the onset of conduction disorders. The most common is left bundle branch block, with a frequency of between 7% and 65% depending on the type of valve implanted. These disorders vary in clinical significance, ranging from spontaneous resolution within a reasonable time to requiring a PM, some of which are urgent (3%–36%) (53). In this respect, Tsoi et al. (54) observed that conduction abnormalities and the need to implant a pacemaker continue to be a frequent and important consequence of this type of replacement. Furthermore, it is important to realise that patients whose initial electrocardiogram reveals pre-existing cardiac conduction abnormalities are at greater risk of needing a pacemaker following TAVI.

Many patients with PM present comorbidities or have undergone previous interventions, and this must be considered when considering possible complications. Although pacemakers are used to enhance the patient's haemodynamic status, Zhang et al. (55) showed that implantation can also provoke coagulation-related complications. Therefore, it is important to consider whether the patient is on antithrombotic or anticoagulant therapy, since this heightens vulnerability to complications related to bleeding, such as haematomas and thrombi.

Haemodialysis patients are more likely to need a pacemaker for bradyarrhythmia treatment. In order to perform dialysis, it is very important to maintain venous circulation. Maradey et al. (56) reported that the first short-term studies of Micra devices have detected an increase in venous access complications, cardiac perforation and pericardial effusions, compared to traditional methods. Nevertheless, initial results are encouraging, as these devices provide a relatively safe long-term pacemaker option for patients who have complex access problems, or who are on haemodialysis.

As with all surgical interventions, PPI is also subject to certain rare complications. Kim et al. (57) highlighted one such. In this case, the PM was inserted into a neonate, and the generator migrated, causing a perforation of the intestine. This occurred because the tissue could not support the weight of the generator, and it was dislodged from its original position into the intraperitoneal space. This sort of occurrence should be suspected in response to gastrointestinal symptoms such as abdominal pain, diarrhoea or fever.

In paediatric patients, therefore, the optimum location for the generator pocket is in the rectus abdominis sheath, which is sometimes reinforced with polytetrafluoroethylene patches. In another case report, the patient presented with severe pain in the area of the pacemaker pocket and was discovered to have experienced a non-traumatic or stress fracture of the second rib, which was causing the pain. This is a rare complication, but it can also be provoked by repetitive mechanical stress on the rib from movement of the device (58).

As a future line of research, the lack of patient knowledge about PPM should be highlighted. Complications may extend far beyond the postoperative period. In fact, some authors point out that patients want to be informed about all relevant aspects of the therapy at the time of implantation (59). This could be very useful, as knowledge about the functionality of PPM may prevent distress among patients and their relatives.

A major limitation in current studies related to PPI is the great variability of indications and of procedures and insertion techniques employed, many of which depend on the experience of the surgical team. Moreover, many investigations do not consider whether the complications observed are related to the type of venous access used in the surgery, although this question is relevant to the duration of the intervention, the area exposed, etc. In addition, few studies have analysed in detail the possible complications of PPI, and these few have been conducted in a small number of countries. We should also note that most of the evidence was observational and derived from a limited number of countries. Furthermore, rare but clinically serious complications were only recorded in case reports, meaning that prevalence estimates are unreliable.

Finally, the contemporary analysis of postsurgical complications is complex. This study focuses on short- and medium-term complications, with little evidence on the long-term performance of PPM. In addition, problems and complications related to the type of stimulation evaluated are now analysed. This means that the typical and well-known problems remain in the background and there is less literature on the subject.

## 5 Conclusions

Pacemaker implantation is a common procedure, but like any surgical intervention it is subject to potential complications, the most common of which is infection. Infections can appear quickly or even years later. Furthermore, they can be complicated by failure to remove the CIED or its electrodes.

Furthermore, many patients with a pacemaker present comorbidity that can worsen the prognosis or increase the chances of complications. The most common comorbidities are related to other diseases (cardiovascular, renal or haematological), the implantation technique used or the device electrodes. Less common comorbidities included pneumothorax, haemorrhages, shoulder pain, and phlebitis, among others.

For this reason, it is essential to minimise the risks, both during and after the procedure and to identify any signs and symptoms that might indicate some type of device complication. In addition, it is important that the device be matched to the patient's characteristics and medical history, thus optimising the treatment and improving the quality of life.

## Data Availability

The raw data supporting the conclusions of this article will be made available by the authors, without undue reservation.
